# 95818 Physical and Cognitive Resilience and Alzheimer’s Disease in a Tibetan Buddhist Monastic Community

**DOI:** 10.1017/cts.2021.729

**Published:** 2021-03-30

**Authors:** Tenzin Namdul, Richard MacLehose, Dedra Buchwald

**Affiliations:** 1 University of Minnesota; 2 Washington State University

## Abstract

ABSTRACT IMPACT: The findings of this study could lend us insights into behavioral intervention that could potentially prevent or slow the onset of Alzheimer’s disease. OBJECTIVES/GOALS: This study examines the association between cognitive and physical resilience and Alzheimer’s disease in a Tibetan Buddhist monastic community in southern India. METHODS/STUDY POPULATION: The study will employ mixed methods of semi- and unstructured interviews and surveys. The interviews will be conducted among 60 monks of age 50+ in six Tibetan monastic colleges in southern India. The interviews will comprise general questions related to monks’ monastic educations and practices, as well as clinical cognitive interviews. Interviewees will be randomly sampled from a census of monks at the six monasteries. Owing to COVID-19 crisis, we will begin data collection, starting with interviews via zoom in mid-December 2020. The survey, which includes demographic information, cognitive assessments, meditative practices, health, memory and physical activity, will be conducted among 400 monks. The survey will be performed onsite and is tentatively scheduled in the summer of 2021. RESULTS/ANTICIPATED RESULTS: The study will help to identify factors associated with physical and cognitive resilience and develop measures to quantify and describe meditative and cognitive practices. These data will be used to both adapt validated measures developed in Western populations for use with this unique population and to develop new items on physical and cognitive resilience to include in the planned survey. Furthermore, the study will provide information about the prevalence of Mild Cognitive Impairment and Alzheimer’s disease and related dementias in this population and development of the survey to capture culturally appropriate measures, including on meditation. The findings could eventually lend us insights into behavioral intervention that could potentially prevent or slow the onset of Alzheimer’s in wider population. DISCUSSION/SIGNIFICANCE OF FINDINGS: Cognitive and physical resilience are understood to confer significant benefits to health outcomes and healthy aging. However, the factors related to resilience, particularly in older adults, are poorly understood. This study will estimate the link between frequency and intensity of meditative practices and physical and cognitive resilience.

